# The Metaplastic Conundrum: A National Cancer Database Analysis of Metaplastic versus Triple-Negative Ductal Breast Cancer

**DOI:** 10.1245/s10434-025-17932-3

**Published:** 2025-07-29

**Authors:** Catherine G. Pratt, Paul H. McClelland, Szu-Aun Long, Allison N. Moore, Jaime D. Lewis, Alicia A. Heelan

**Affiliations:** 1 Cincinnati Research in Outcomes and Safety in Surgery (CROSS) Research Group, Department of Surgery, University of Cincinnati College of Medicine, Cincinnati, OH USA; 2 Division of Surgical Oncology, Department of Surgery, University of Cincinnati College of Medicine, Cincinnati, OH USA

**Keywords:** Metaplastic breast cancer, Triple-negative metaplastic breast cancer, Biomarker-positive metaplastic breast cancer, Breast cancer

## Abstract

**Background:**

Historically, treatment approaches for metaplastic breast cancer (MpBC) have mirrored that of triple-negative ductal breast cancer (TN-IDC), yet MpBC has persistently worse survival. This study describes rates and response to neoadjuvant systemic therapy (NAC) for MpBC and evaluates survival between triple-negative MpBC (TN-MpBC), biomarker-positive MpBC (nTN-MpBC), and TN-IDC by treatment approach.

**Methods:**

The National Cancer Database was queried for females diagnosed with clinically non-metastatic MpBC or TN-IDC from 2011 to 2021. One-to-one propensity score matching between TN-MpBC and TN-IDC patients was performed.

**Results:**

Of 5575 MpBC patients, surgery and chemotherapy rates were high. For patients who received systemic therapy and surgery, an adjuvant approach was favored; however, NAC for TN-MpBC increased from 18.3 to 31.5% by 2021. Higher rates of NAC non-response and lower overall survival were found among MpBC cohorts compared with TN-IDC. For MpBC, an adjuvant approach had significantly better survival than other systemic therapy sequences. When evaluated by pathologic response to NAC, a partial or non-response had worse survival compared with complete response or not undergoing NAC. On Cox proportional hazard regression of matched patients, NAC had an adjusted hazard ratio of 2.56 (1.36–4.79) compared with not undergoing NAC.

**Conclusion:**

MpBC is predominantly treated with surgery and systemic therapy, with increasing rates of NAC for TN-MpBC. However, patients with MpBC have inferior survival to TN-IDC, and NAC for MpBC is associated with worse survival compared with other systemic therapy sequences, unless a complete pathologic response is achieved. These findings reinforce the need for systemic treatment sequence optimization for MpBC.

**Supplementary Information:**

The online version contains supplementary material available at 10.1245/s10434-025-17932-3.

Metaplastic breast cancer (MpBC) is a rare subset of breast cancers that make up just 0.2–5% of cases.^1–4^ Proven to be both clinically aggressive and treatment resistant, MpBC serves as a persistent therapeutic challenge for cancer care providers.^2,4–10^ Given that breast cancer remains the most common cancer diagnosis among women, even rare subtypes contribute to significant morbidity and mortality. An estimated 317,000 new breast cancer diagnoses are anticipated in the United States (US) alone in 2025,^11^ of which between 634 and 15,850 cases will be metaplastic in nature.

Due to the relative rarity of MpBC, treatment approaches have largely been dictated by studies conducted in other types of breast cancer. The National Comprehensive Cancer Network (NCCN) guidelines include MpBC within the general category of invasive breast cancer and recommend treatment considerations mirroring that of invasive breast cancers with ductal, lobular, mixed, and micropapillary histology.^12^ MpBC behavior has been particularly likened to that of triple-negative ductal breast cancer (TN-IDC) resulting in the adoption of similar treatment strategies.^13–17^ Despite conformity to TN-IDC treatment principles, MpBC has persistently worse survival, even in the age of modern systemic therapeutics.^5,10,14,17–22^ Additionally, and unlike TN-IDC, MpBC has proven resistant to neoadjuvant systemic therapy (NAC), with low rates of pathologic complete response (pCR), high rates of non-response, and even disease progression while receiving NAC.^23–26^ Furthermore, while MpBC is most commonly triple-negative (TN-MpBC) disease, with a lack of expression of the estrogen receptor, the progesterone receptor, and human epidermal growth factor receptor 2 (HER2); some metaplastic cases are biomarker-positive (nTN-MpBC). This further complicates the treatment algorithm from that of an TN-IDC approach.

The use of NAC in breast cancer has expanded substantially from its initial application in inflammatory and locally advanced disease to patients with early-stage tumors, especially HER-2 positive and TN-IDC tumors. Prior literature has demonstrated that some providers are offering NAC for MpBC, however rates of national use and pathologic response remain unclear. Additionally, a paucity of literature describes treatment and outcome differences for TN-MpBC compared with nTN-MpBC. The purpose of this study was to employ a nationwide, multicenter database to (1) assess trends in the application of and response to NAC for MpBC; and (2) evaluate survival between TN-MpBC, nTN-MpBC, and TN-IDC by treatment approach. We hypothesized that an increasing rate of NAC would be employed for MpBC, but with low pCR. We further hypothesized that lower survival would be identified among MpBC cohorts compared with TN-IDC, with NAC non-responders suffering from the lowest survival.

## Methods

### Data Source and Study Population

Data from the National Cancer Database (NCDB) were utilized for this study. A joint initiative between the American College of Surgeons’ and American Cancer Society, the NCDB is a nationwide oncology database including over 1500 Commission on Cancer (CoC)-accredited facilities and capturing 70% of newly diagnosed cancer cases within the US. The NCDB reports data on facility characteristics, patient demographics, tumor characteristics, treatments, and outcomes.^27,28^ Data from the NCDB are de-identified, thus this work was deemed exempt from Institutional Review Board review (IRB#2024-1175).

The NCDB was queried for all females diagnosed with clinically non-metastatic, unilateral MpBC or TN-IDC from 2011 to 2021. Patients with incomplete data regarding biomarker status, clinical staging, treatment(s) received, and/or treatment sequence were excluded. Additional exclusion criteria included patients who were coded in the database as having refused any portion of the physician-recommended treatment course (surgery, chemotherapy, immunotherapy, hormone therapy, or radiation). Patients were grouped and analyzed based on histology and biomarker status: TN-MpBC, nTN-MpBC, or TN-IDC.

### Data Variables

Baseline characteristics examined included age, race, ethnicity, treatment facility type, insurance status, geographical location size, distance from treatment facility, and Charlson–Deyo Comorbidity Index. Cancer-specific factors included clinical tumor, node, metastasis (TNM) stage, clinical grade, Ki-67, lymphovascular invasion, tumor size, regional lymph node positivity, and pathologic TNM stage. Examined treatment and outcome variables included type of treatment (surgery, chemotherapy, immunotherapy, hormone therapy, and/or radiation), treatment sequence, type of surgical resection (partial mastectomy, mastectomy, or bilateral mastectomy), residual tumor on pathological report following resection, regional nodes examined, regional lymph node surgery, type of chemotherapy (multi-agent or single-agent), response to NAC (pCR, partial response, or non-response), 30-day mortality, 90-day mortality, and overall mortality. These factors and temporal trends in management approach for MpBC were evaluated.

### Statistical Analysis

Continuous variables were compared using the Wilcoxon rank-sum test, and Pearson’s Chi-square tests were utilized for analysis of categorical variables. All tests of significance were two-tailed, with a *p*-value < 0.05 indicating statistical significance. Continuous variables were described using median [interquartile range, and categorical variables were summarized using frequencies and percentages as appropriate. If incomplete data were noted for a case entry, that case was excluded from the specific analysis for which data were unavailable. Overall survival (OS) for patients were compared using Kaplan–Meier analysis, with log-rank *p*-values reported. OS was defined as time from the date of diagnosis until the date of reported death or last documented patient contact. Linear regression was used to evaluate systemic therapy sequencing and rate of pathologic response to NAC over time. One-to-one propensity score matching between TN-MpBC and TN-IDC patients was performed based on age, race, ethnicity, treatment facility type, insurance status, geographical location size, distance from treatment facility, clinical TNM stage, clinical grade, Ki-67, lymphovascular invasion, tumor size, type of surgical resection, residual tumor following resection, regional lymph node positivity, systemic therapy received, radiation therapy received, pathologic TNM stage, and pathologic stage group. Standardized mean differences (SMD) and variance ratios were calculated using pooled standard deviations to estimate average treatment effects, which assumes no bias towards either matched group, as validated in previous literature.^29^ Cox proportional hazard regression modeling was performed on the matched patients to identify factors associated with mortality. Hazard regression results are reported as adjusted hazard ratios (aHR) with 95% confidence intervals. Factors with a *p*-Value < 0.2 on univariable modeling were utilized as initial covariates for multivariable regression analysis. All statistical analyses were performed using JMP Pro v18 (SAS Institute, Inc., Cary, NC, USA) and R statistical software v4.3.1 (R Foundation for Statistical Computing, Vienna, Austria; 16 June 2023).

## Results

### Metaplastic Breast Cancer

A total of 7616 female patients with clinically non-metastatic, unilateral MpBC on histology were identified. 1691 patients had missing data in one or more category regarding biomarker status, treatment completion, treatment sequencing, or declining at least one element of their physician-recommended treatment and were subsequently excluded from the analysis. Of the remaining 5575 patients who met the full inclusion criteria, 74.9% (*n* = 4175) had triple-negative disease and 25.1% (*n* = 1400) were positive for at least one biomarker. The overall baseline demographics for the cohort with MpBC, and a comparison between TN-MpBC and nTN-MpBC are outlined in electronic supplementary material (ESM) Table 1. Most patients were White race (77.2%), treated in a metro geographic location, and had a Charlson–Deyo Comorbidity Index of 0. On comparison between MpBC groups, only age and treatment facility type were different. Compared with nTN-MpBC patients, those with TN-MpBC were slightly older (median age 63 vs. 61 years; *p* < 0.0001), more likely to be treated at an academic/research program (34.2% vs. 30.9%; *p* = 0.01), and less likely to be treated at a comprehensive community cancer program (37.0% vs. 41.4%; *p* = 0.01).

When evaluating cancer-specific factors, most MpBC patients had clinical T1 (31.9%) or T2 (48.7%) and clinical N0 (83.4%) disease, with poorly or undifferentiated clinical grade (82.9%) (Table 1). Pathologic TNM stages were most frequently T1 (34.1%) or T2 (46.7%), N0 (82.0%), and M0 (98.9%). On comparison between groups, the distribution of patients presenting with clinical T stage were equivalent, however nTN-MpBC patients were less likely to have clinical N0 (84.5% vs. 80.2%) and more likely to have clinical N1 (12.3% vs. 16.0%) disease (*p* = 0.001). nTN-MpBC patients also had lower rates of moderately differentiated clinical grade (14.7% vs. 13.9%; *p* < 0.0001) with higher rates of lymphovascular invasion (14.5% vs. 17.7%; *p* = 0.01) and regional lymph node positivity (17.1% vs. 24.5%; *p* < 0.0001) than TN-MpBC patients. Pathologic TNM stage was more likely to be T0 (2.2% vs. 3.3%) or T2 (45.9% vs. 49.2%) [*p* = 0.0009] and N1 (12.6% vs. 18.0%; *p* < 0.0001) in the nTN-MpBC group, but more likely T3 (13.2% vs. 9.6%) or T4 (4.8% vs. 3.5%) [*p* = 0.0009] and N0 (82.0% vs. 77.8%; *p* < 0.0001) for TN-MpBC patients.

**Table 1:** Cancer-specific factor comparison between triple-negative and biomarker-positive metaplastic breast cancer

Category	Overall [*N* = 5575]	TN-MpBC [*n* = 4175 (74.9%)]	nTN-MpBC [*n* = 1400 (25.1%)]	*p*-Value
Clinical T stage				0.3
cT1	1780 (31.9)	1341 (32.1)	439 (31.3)	
cT2	2713 (48.7)	2003 (48.0)	710 (50.7)	
cT3	721(12.9)	555 (13.3)	166 (11.9)	
cT4	361 (6.5)	276 (6.6)	85 (6.1)	
Clinical N stage				**0.001**
cN0	4605 (83.4)	3492 (84.5)	1113 (80.2)	
cN1	729 (13.2)	507 (12.3)	222 (16.0)	
cN2	124 (2.3)	93 (2.3)	31 (2.2)	
cN3	65 (1.2)	43 (1.0)	22 (1.6)	
Clinical M0 stage	5575 (100)	4175 (100)	1400 (100)	0.99
Clinical grade				**< 0.0001**
Well-differentiated	146 (2.6)	107 (2.6)	39 (2.8)	
Moderately differentiated	809 (14.5)	615 (14.7)	194 (13.9)	
Poorly/undifferentiated	4620 (82.9)	3543 (82.7)	1167 (83.4)	
Ki-67, % (median [IQR])	55 [30–80]	54 [30–80]	60 [30–80]	0.07
Lymphovascular invasion	705 (15.3)	501 (14.5)	204 (17.7)	**0.01**
Tumor size, mm (median [IQR])	30 [19–45]	30 [19–46]	30 [19–43]	0.1
Regional lymph node positivity	2057 (17.8)	713 (17.1)	344 (24.5)	**< 0.0001**
Pathologic T stage				**0.0009**
pT0	114 (2.5)	76 (2.2)	38 (3.3)	
pT1	1567 (34.1)	1196 (34.0)	398 (34.4)	
pT2	2147 (46.7)	1578 (45.9)	569 (49.2)	
pT3	564 (12.3)	453 (13.2)	111 (9.6)	
pT4	206 (4.5)	166 (4.8)	40 (3.5)	
Pathologic N stage				**< 0.0001**
pN0	3578 (82.0)	2716 (83.4)	862 (77.8)	
pN1	609 (14.0)	410 (12.6)	199 (18.0)	
pN2	130 (3.0)	99 (3.0)	31 (2.8)	
pN3	48 (1.1)	32 (1.0)	16 (1.4)	
Pathologic M0 stage	2433 (98.9)	1787 (98.9)	646 (98.8)	0.99

Bold values indicate statistical significance *p*-value < 0.5

Data are expressed as *n* (%) unless otherwise specified

*nTN-MpBC* biomarker-positive metaplastic breast cancer, *TN-MpBC* triple-negative metaplastic breast cancer, *IQR* interquartile range

Among treatment and outcomes variables evaluated, the majority of MpBC patients received surgical resection (97.3%), had regional lymph nodes examined (91.1%), underwent chemotherapy (79.8%), and received radiation therapy (58.3%) (Table 2). Between-group comparison revealed higher rates of residual tumor following surgery (3.3% vs. 2.2%; *p* = 0.04) for TN-MpBC, but a similar distribution of surgical approach to the primary tumor for both groups. Among patients who received chemotherapy as part of their treatment regimen, TN-MpBC patients were more frequently treated with a multi-agent chemotherapy regimen (95.8% vs. 93.9%; *p* = 0.04) and had higher rates of NAC sequencing (20.2% vs. 13.4%; *p* < 0.0001) compared with nTN-MpBC. However, TN-MpBC patients also saw more frequent use of surgical resection alone without systemic therapy (19.0% vs. 9.6%), whereas nTN-MpBC patients were more commonly sequenced with an adjuvant approach (51.3% vs. 61.7%) or both neoadjuvant and adjuvant systemic therapy (9.4% vs. 15.3%) [*p* < 0.0001]. The nTN-MpBC group saw more utilization of immunotherapy (4.1% vs. 13.5%; *p* < 0.0001) and hormone therapy (2.5% vs. 51.9%; *p* < 0.0001) than the TN-MpBC group. Pathologic response to NAC was similar between MpBC cohorts with low rates of pCR (15.8% vs. 20.1%; *p* = 0.2). Thirty-day mortality was equivalent between MpBC groups, but 90-day (1.5% vs. 0.4%; *p* = 0.003) and overall mortality (30.6% vs. 27.4%; *p* = 0.04) were worse for TN-MpBC patients than nTN-MpBC patients, at a median follow-up of 51.1 months. On Kaplan–Meier analysis, there was no significant difference in OS between the TN-MpBC and nTN-MpBC groups.

**Table 2:** Treatment and outcome factor comparison between triple-negative and biomarker-positive metaplastic breast cancer

Category	Overall [*n* = 5575]	TN-MpBC [*n* = 4175 (74.9%)]	nTN-MpBC [*n* = 1400 (25.1%)]	*p*-Value
Received surgical resection	5423 (97.3)	4070 (97.5)	1353 (96.6)	0.09
Extent of surgical resection				0.6
Partial mastectomy	2433 (44.9)	1841 (45.3)	592 (43.9)	
Mastectomy	2189 (40.4)	1630 (40.1)	559 (41.4)	
Bilateral mastectomy	793 (14.6)	594 (14.6)	199 (14.7)	
Residual tumor	164 (3.0)	134 (3.3)	30 (2.2)	**0.04**
Regional lymph nodes examined	5081 (91.1)	3799 (91.0)	1282 (91.6)	0.5
Regional lymph node surgery				0.7
Biopsy only	41 (0.8)	29 (0.8)	12 (0.9)	
Sentinel lymph node biopsy	3054 (60.1)	2297 (60.4)	757 (59.1)	
Axillary lymph node dissection	840 (16.5)	586 (14.8)	212 (16.1)	
Sentinel lymph node biopsy and axillary lymph node dissection	766 (15.1)	572 (15.1)	194 (15.2)	
Unspecified	380 (7.5)	289 (7.6)	91 (7.1)	
Received chemotherapy	4446 (79.8)	3343 (80.1)	1103 (78.8)	0.3
Multi-agent chemotherapy	4238 (95.3)	3202 (95.8)	1036 (93.9)	**0.04**
Received immunotherapy	361 (6.5)	172 (4.1)	189 (13.5)	**< 0.0001**
Received hormone therapy	832 (14.9)	106 (2.5)	726 (51.9)	**< 0.0001**
Systemic therapy sequence				**< 0.0001**
Neoadjuvant	1004 (18.5)	823 (20.2)	181 (13.4)	
Adjuvant	2924 (53.9)	2089 (51.3)	835 (61.7)	
Neoadjuvant and adjuvant	590 (10.8)	383 (9.4)	207 (15.3)	
Surgery alone	904 (16.7)	774 (19.0)	130 (9.6)	
Received radiation	3251 (58.3)	2435 (58.3)	816 (58.3)	0.99
Radiation therapy sequence				
Neoadjuvant	90 (0.4)	23 (1.0)	7 (0.9)	0.5
Adjuvant	3189 (98.8)	2387 (98.8)	802 (98.7)	
Neoadjuvant and Adjuvant		6 (0.3)	4 (0.5)	
Response to NAC				0.2
Complete response	213 (16.8)	152 (15.8)	61 (20.1)	
Partial response	427 (33.7)	324 (33.6)	103 (34.0)	
Non-response	299 (23.6)	228 (23.6)	71 (23.4)	
30-day mortality	13 (0.3)	12 (0.3)	1 (0.1)	0.1
90-day mortality	58 (1.2)	53 (1.5)	5 (0.4)	**0.003**
Overall mortality	1487 (29.8)	1139 (30.6)	348 (27.4)	**0.04**
Median time to last follow-up, months (median [IQR])	51.1 [26.5–81.8]	51.3 [26.4–82.7]	51.0 [27.3–79.8]	0.4

Bold values indicate statistical significance *p*-value < 0.5

Data are expressed as *n* (%) unless otherwise specified

*NAC* neoadjuvant systemic therapy, *nTN-MpBC* biomarker-positive metaplastic breast cancer, *TN-MpBC* triple-negative metaplastic breast cancer, *IQR* interquartile range

**Fig. 1:**
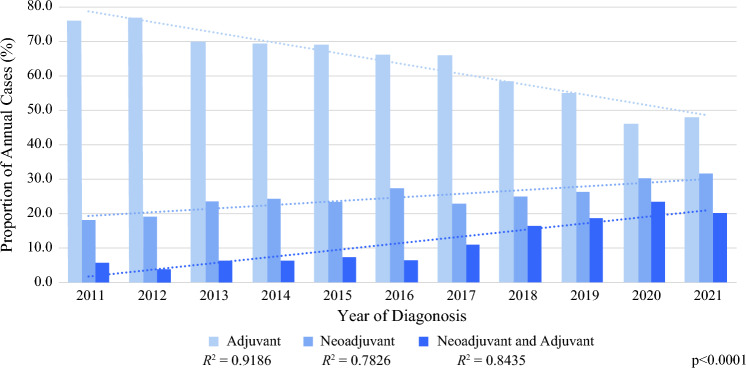
Rates of systemic treatment sequence among triple-negative metaplastic breast cancer patients by year of diagnosis

When evaluating annual trends in treatment sequencing for systemic therapy and surgery, the rate of NAC and combined neoadjuvant and adjuvant systemic therapy increased annually for TN-MpBC patients, while the rate of adjuvant systemic therapy decreased (Fig. 1). Over the study period, NAC among patients who received systemic therapy increased from 18.3 to 31.5% of cases, combined neoadjuvant and adjuvant systemic therapy increased from 5.7 to 20.1%, and adjuvant systemic therapy decreased from 76.0 to 48.0% of cases. On linear regression analysis, year of diagnosis significantly predicted the systemic therapy sequencing rate (adjuvant R^2^ = 0.92, neoadjuvant R^2^ = 0.78, combined neoadjuvant and adjuvant R^2^ = 0.84). No linear relationship was identified for the annual rate of systemic treatment sequence for nTN-MpBC or the annual rate of TN-MpBC patients who received surgery alone without systemic therapy.

When evaluating annual trends in the pathologic response to NAC among patients with MpBC, no significant difference was found in the proportion of pathologic non-response versus response (partial or pCR) over time for TN-MpBC (R^2^ = 0.03; *p* = 0.6). Similarly, when evaluating the annual rate of pCR versus partial pathologic response, no significant change was identified by year of diagnosis (pCR: R^2^ = 0.16, *p* = 0.2; partial response: R^2^ = 0.27, *p* = 0.1). A similar lack of change over time among nTN-MpBC patients was found for overall response and pCR rates.

### Metaplastic versus Triple-Negative Ductal Breast Cancer

A total of 144,080 patients with TN-IDC were identified. The baseline demographic comparison between TN-MpBC (*n* = 4175, 2.8%), nTN-MpBC (*n* = 1400, 0.9%), and TN-IDC (*n* = 144,080, 96.3%) is outlined in ESM Table 2. The MpBC cohorts were older and more likely White race, treated at an academic/research program, and insured with government insurance, and less likely Black race or to have a Charlson–Deyo Comorbidity Index of 0 than the TN-IDC group.

When evaluating cancer-specific factors, both MpBC cohorts had higher clinical and pathologic T stages (*p* < 0.0001) (Table 3). Conversely, clinical and pathologic N stages were lower for both TN-MpBC and nTN-MpBC compared with TN-IDC (*p* < 0.0001). TN-IDC had a higher likelihood of moderate differentiation on clinical grade, lymphovascular invasion, and regional lymph node positivity, and higher rates of Ki-67 than either group with MpBC (*p* < 0.0001 for all).

**Table 3:** Cancer-specific factor comparison between triple-negative metaplastic breast cancer, biomarker-positive metaplastic breast cancer, and triple-negative ductal breast cancer

Category	TN-MpBC [*n* = 4175 (2.8%)]	nTN-MpBC [*n* = 1400 (0.9%)]	TN-IDC [*n* = 144,080 (96.3%)]	*p*-Value
Clinical T stage				**< 0.0001**
cT1	1341 (32.1)	439 (31.3)	68,191 (47.3)	
cT2	2003 (48.0)	710 (50.7)	58,836 (40.8)	
cT3	555 (13.3)	166 (11.9)	11,221 (7.8)	
cT4	276 (6.6)	85 (6.1)	5832 (4.1)	
Clinical N stage				**< 0.0001**
cN0	3492 (84.5)	1113 (80.2)	105,605 (73.9)	
cN1	507 (12.3)	222 (16.0)	29,218 (20.5)	
cN2	93 (2.3)	31 (2.2)	4385 (3.1)	
cN3	43 (1.0)	22 (1.6)	3677 (2.6)	
Clinical M0 stage	4175 (100)	1400 (100)	144,080 (100)	0.99
Clinical grade				**< 0.0001**
Well differentiated	107 (2.6)	39 (2.8)	2164 (1.5)	
Moderately differentiated	615 (14.7)	194 (13.9)	25,218 (17.5)	
Poorly/undifferentiated	3543 (82.7)	1167 (83.4)	116,698 (81.0)	
Ki-67, % (median [IQR])	54 [30–80]	60 [30–80]	65 [40–81]	**< 0.0001**
Lymphovascular invasion	501 (14.5)	204 (17.7)	23,201 (20.6)	**< 0.0001**
Tumor size, mm (median [IQR])	30 [19–46]	30 [19–43]	22 [13–33]	**< 0.0001**
Regional lymph node positivity	713 (17.1)	344 (24.5)	37,431 (26.0)	**< 0.0001**
Pathologic T stage				**< 0.0001**
pT0	76 (2.2)	38 (3.3)	9779 (9.5)	
pT1	1196 (34.0)	398 (34.4)	59,390 (57.6)	
pT2	1578 (45.9)	569 (49.2)	28,951 (28.1)	
pT3	453 (13.2)	111 (9.6)	3578 (3.5)	
pT4	166 (4.8)	40 (3.5)	1396 (1.4)	
Pathologic N stage				**< 0.0001**
pN0	2716 (83.4)	862 (77.8)	79,221 (77.2)	
pN1	410 (12.6)	199 (18.0)	17,058 (16.6)	
pN2	99 (3.0)	31 (2.8)	4409 (4.3)	
pN3	32 (1.0)	16 (1.4)	1982 (1.9)	
Pathologic M0 stage	1787 (98.9)	646 (98.8)	51,996 (99.5)	**0.0002**

Bold values indicate statistical significance *p*-value < 0.5

Data are expressed as *n* (%) unless otherwise specified

*nTN-MpBC* biomarker-positive metaplastic breast cancer, *TN-IDC* triple-negative ductal breast cancer, *TN-MpBC* triple-negative metaplastic breast cancer, *IQR* interquartile range

Among treatment and outcomes variables evaluated, TN-MpBC patients had slightly higher rates of surgical resection (97.5%) than nTN-MpBC (96.6%) or TN-IDC patients (96.1%) [*p* < 0.0001]) but were more likely to have residual tumor after resection (3.3% vs. 2.2% vs. 2.6%; *p* = 0.02) (Table 4). Both the TN-MpBC (45.3%) and nTN-MpBC (43.9%) cohorts were less likely to receive a partial mastectomy than the TN-IDC cohort (53.2%) [*p* < 0.0001] or have regional lymph nodes examined (91.0% vs. 91.6% vs. 93.4%; *p* < 0.0001). Compared with TN-MpBC and nTN-MpBC, TN-IDC patients were more likely to receive chemotherapy (80.1% vs. 78.8% vs. 87.1%; *p* < 0.0001) and radiation therapy (58.3% vs. 58.3% vs. 63.4%; *p* < 0.0001) and had a higher likelihood of an NAC sequence of systemic therapy (20.2% vs. 13.4% vs. 32.9%; *p* < 0.0001). nTN-MpBC patients had a lower likelihood of receiving a multi-agent chemotherapy regimen (95.8% vs. 93.9% vs. 95.7%; *p* = 0.002), but more were more likely to receive immunotherapy (4.1% vs. 13.5% vs. 5.3%; *p* < 0.0001) and hormone therapy (2.5% vs. 51.9% vs. 3.0%; *p* < 0.0001) than triple-negative patients in either group. When considering response to NAC, higher rates of non-response were found among TN-MpBC (23.6%) and nTN-MpBC (23.4%) patients compared with TN-IDC (6.5%) [*p* < 0.0011]. 30- and 90-day mortality was higher among TN-MpBC (*p* = 0.0002 and *p* < 0.0001). At a median follow-up time of 51 months for MpBC and 54 months for TN-IDC, overall mortality was higher for TN-MpBC (30.6%) and nTN-MpBC (27.4%) compared with TN-IDC (20.4%) [*p* < 0.0001).

**Table 4:** Treatment and outcome factor comparison between triple-negative metaplastic breast cancer, biomarker-positive metaplastic breast cancer, and triple-negative ductal breast cancer

Category	TN-MpBC [*n* = 4175 (2.8%)]	nTN-MpBC [*n* = 1400 (0.9%)]	TN-IDC [*n* = 144,080 (96.3%)]	*p*-Value
Received surgical resection	4070 (97.5)	1353 (96.6)	138,453 (96.1)	**< 0.0001**
Extent of surgical resection				**< 0.0001**
Partial mastectomy	1841 (45.3)	592 (43.9)	73,573 (53.2)	
Mastectomy	1630 (40.1)	559 (41.4)	37,847 (26.4)	
Bilateral mastectomy	594 (14.6)	199 (14.7)	26,953 (19.5)	
Residual tumor	134 (3.3)	30 (2.2)	3567 (2.6)	**0.02**
Regional lymph nodes examined	3799 (91.0)	1282 (91.6)	134,582 (93.4)	**< 0.0001**
Regional lymph node surgery				0.2
Biopsy only	29 (0.8)	12 (0.9)	1576 (1.2)	
Sentinel lymph node biopsy	2297 (60.4)	757 (59.1)	79,912 (59.4)	
Axillary lymph node dissection	586 (14.8)	212 (16.1)	22,621 (16.8)	
Sentinel lymph node biopsy and axillary lymph node dissection	572 (15.1)	194 (15.2)	19,678 (14.6)	
Unspecified	289 (7.6)	91 (7.1)	10,778 (8.0)	
Received chemotherapy	3343 (80.1)	1103 (78.8)	125,476 (87.1)	**< 0.0001**
Multi-agent chemotherapy	3202 (95.8)	1036 (93.9)	120,051 (95.7)	**0.002**
Received immunotherapy	172 (4.1)	189 (13.5)	7568 (5.3)	**< 0.0001**
Received hormone therapy	106 (2.5)	726 (51.9)	4316 (3.0)	**< 0.0001**
Systemic therapy sequence				**< 0.0001**
Neoadjuvant	823 (20.2)	181 (13.4)	45,495 (32.9)	
Adjuvant	2089 (51.3)	835 (61.7)	63,555 (45.9)	
Neoadjuvant and adjuvant	383 (9.4)	207 (15.3)	13,843 (10.0)	
Surgery alone	774 (19.0)	130 (9.6)	15,560 (11.2)	
Received radiation	2435 (58.3)	816 (58.3)	91,342 (63.4)	**< 0.0001**
Radiation therapy sequence				0.7
Neoadjuvant	23 (1.0)	7 (0.9)	714 (0.8)	
Adjuvant	2387 (98.8)	802 (98.7)	89,798 (98.8)	
Neoadjuvant and adjuvant	6 (0.3)	4 (0.5)	336 (0.4)	
Response to NAC				**< 0.0001**
Complete Response	152 (15.8)	61 (20.1)	19,851 (40.4)	
Partial Response	324 (33.6)	103 (34.0)	14,626 (29.7)	
Non-Response	228 (23.6)	71 (23.4)	3189 (6.5)	
30-day mortality	12 (0.3)	1 (0.1)	128 (0.1)	**0.0002**
90-day mortality	53 (1.5)	5 (0.4)	592 (0.5)	**< 0.0001**
Overall mortality	1139 (30.6)	348 (27.4)	26,195 (20.4)	**< 0.0001**
Median time to last follow-up, months (median [IQR])	51.3 [26.4–82.7]	51.0 [27.3–79.8]	54.0 [31.0–85.6]	**< 0.0001**

Bold values indicate statistical significance *p*-value < 0.5

Data are expressed as *n* (%) unless otherwise specified

*NAC* neoadjuvant systemic therapy, *nTN-MpBC* biomarker-positive metaplastic breast cancer, *TN-IDC* triple-negative ductal breast cancer, *TN-MpBC* triple-negative metaplastic breast cancer, *IQR* interquartile range

**Fig. 2:**
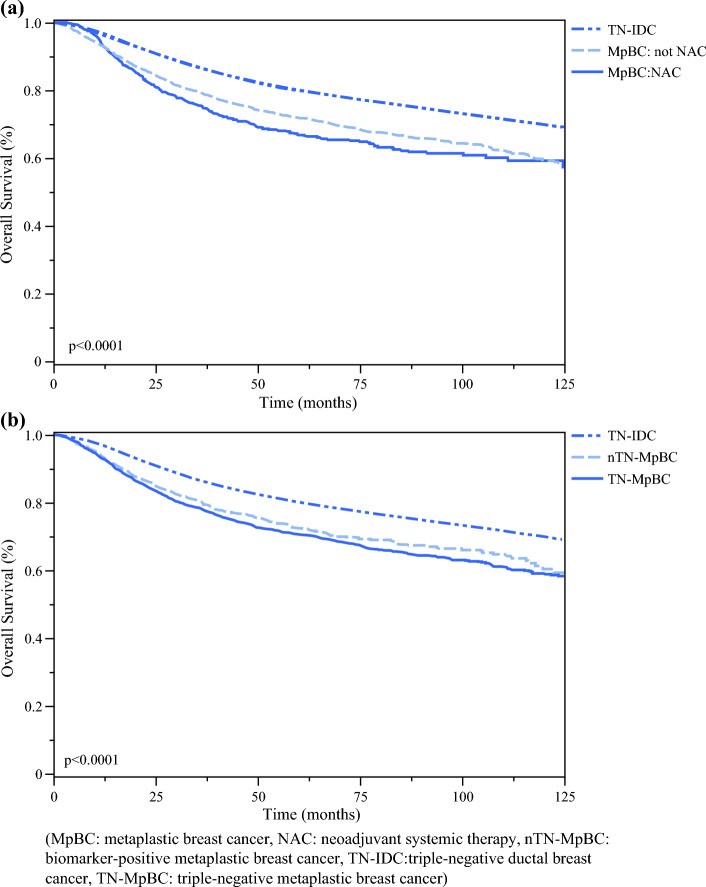
Kaplan–Meier survival curves by (**a**) biomarker status, and (**b**) biomarker status and sequence of systemic therapy.

**Fig. 3:**
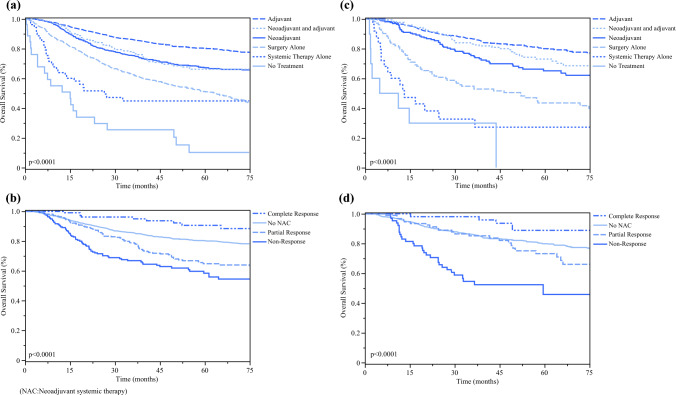
Kaplan–Meier survival curves for triple-negative metaplastic breast cancer comparing overall survival by **a** triple-negative metaplastic breast cancer sequence of systemic therapy; **b** triple-negative metaplastic breast cancer response to neoadjuvant systemic therapy; **c** biomarker-positive metaplastic breast cancer sequence of systemic therapy; and **d** biomarker-positive metaplastic breast cancer response to neoadjuvant systemic therapy. *NAC* neoadjuvant chemotherapy

On Kaplan–Meier survival analysis, TN-MpBC and nTN-MpBC had lower OS than TN-IDC (*p* < 0.0001) (Fig. 2). When OS was evaluated by MpBC undergoing versus not undergoing NAC, an NAC approach for MpBC regardless of biomarker status had significantly worse OS than either not undergoing NAC or TN-IDC (*p* < 0.0001). On sensitivity analysis of survival for MpBC cohorts and sequence of systemic therapy, an adjuvant approach had significantly better OS than all other approaches to treatment, including NAC (*p* < 0.0001) (Fig. 3). When evaluated by pathologic response to NAC, a partial or non-response had worse survival compared with complete response and not undergoing NAC (*p* < 0.0001).

Finally, on Cox proportional hazard regression modeling of the matched patients (*n* = 642, SMD range −0.011 to 0.97, variance ratio range 0.97–1.67), NAC was associated with mortality (aHR 2.56 [2.36–4.79]; *p* = 0.003) compared with not undergoing NAC (ESM Table 3). Clinical T4 stage versus clinical T0 (aHR 8.29 [1.20–57.00]; *p* = 0.03) and regional lymph node positivity versus no positive regional lymph nodes (aHR 6.53 [1.24–34.42]; *p* = 0.03) were also independently associated with mortality on regression modeling.

## Discussion

This NCDB analysis demonstrated a predominance of both surgical resection and systemic therapy as treatment strategies for MpBC, with an increasing utilization of NAC for triple-negative disease. This generally aligns with patterns of treatment for TN-IDC; however, OS for MpBC remains significantly lower than TN-IDC regardless of biomarker status. In accordance with published literature, the rate of pCR following NAC was found to be low for MpBC cohorts and significantly lower compared with the rate for TN-IDC. Finally, an NAC approach for both TN-MpBC and nTN-MpBC was found to be associated with worse OS than not receiving NAC, unless pCR was achieved.

Previous studies evaluating MpBC have demonstrated the heterogenous nature of this subset of breast cancer. Most MpBC is triple-negative, with rapid growth, large tumor size at presentation, and lower rates of axillary lymph node involvement.^5,30,31^ Similarly, our identified MpBC cohort was predominantly triple-negative, clinical T2 and N0 stage, with poorly differentiated or undifferentiated clinical grade, and pathologic T2 and N0 stage. While biomarker positivity, specifically hormone receptor positivity, is considered a good prognostic factor for other types of breast cancer, this has not been found to be true with MpBC^32^. However, few studies have evaluated differences in TN-MpBC compared with nTN-MpBC, particularly when it comes to treatment approach and response to NAC. Our study identified cancer-specific, treatment, and outcome differences when TN-MpBC was considered separately from nTN-MpBC. Notably, TN-MpBC was associated with lower clinical and pathologic nodal stage and regional node positivity, but higher pathologic T stage. Unsurprisingly, nTN-MpBC was more frequently treated with systemic adjuncts of immunotherapy and hormone therapy, but was less likely to receive a multi-agent chemotherapy regimen over a single agent. Despite these differences, OS was similar between MpBC cohorts.

Importantly, our findings demonstrated a similar pattern of pathologic response to NAC to that which has been described in previous studies; however, we also consider results of NAC response among TN-MpBC and nTN-MpBC as separate entities. In a retrospective cohort of MpBC treated at a single center, Wong et al. found a pCR rate of 10–17%, with the majority of NAC patients instead experiencing a poor response or disease progression,^33^ while Han et al. demonstrated a pCR of 17.6%.^34^ Our study, drawn from a national cohort of cancer centers, revealed an overall MpBC pCR rate of 16.8%. More specifically, pCR was achieved among 15.8% of TN-MpBC cases and 20.1% for nTN-MpBC. While slightly higher for nTN-MpBC patients, this response still fell well below the demonstrated rate for TN-IDC, at 40.4%. Additionally, rates of NAC non-response for MpBC cohorts were over threefold higher than that of TN-IDC. For MpBC patients receiving NAC, the likelihood of non-response was equivalent to that of partial response, and the chance of pCR was slim.

Regarding trends in treatment approaches and survival, Miao et al. recently described notable improvements in OS over the past 20 years for TN-MpBC patients that was not demonstrated among an evaluated nTN-MpBC group.^35^ However, while they discuss benefits of systemic treatment in addition to surgical resection, their analysis did not consider the sequence of treatment, nor did it compare survival among biomarker distinct MpBC cohorts. This study demonstrated an increased likelihood of either an NAC approach or surgical resection without any systemic therapy for TN-MpBC compared with nTN-MpBC. Additionally, an increasing rate of NAC and combined neoadjuvant and adjuvant systemic therapy was found over the study period in this cohort with a consistent rate of a surgical resection alone approach. The increase in NAC mirrors increasing utilization in TN-IDC, especially among early-stage disease.^36^ This would suggest that cancer providers continue to consider MpBC as a similar entity to that of TN-IDC when it comes to systemic therapy planning. Where MpBC treatment algorithms appeared to differ from TN-IDC was regarding surgical resection. At a national level, MpBC patients had a higher likelihood of mastectomy (simple or modified radical) over partial mastectomy, which may be explained by the predominance of larger metaplastic tumors when compared with TN-IDC. Conversely, a lower likelihood of regional lymph node evaluation was noted compared with TN-IDC patients.

Unfortunately, we found that any treatment sequence other than adjuvant systemic therapy was associated with worse OS for MpBC, unless pCR was achieved with an NAC approach. While the short- and long-term benefits of pCR following NAC are well established,^37,38^ patients who fail to achieve pCR have increased long-term risk. Previous work has shown that patients with residual breast cancer following NAC are at high risk of relapse, despite completion of a full treatment course (including planned surgery and adjuvant systemic therapy if indicated).^39,40^ This study showed that although MpBC patients not treated with NAC continued to experience worse OS that TN-IDC, survival in this group was significantly better than MpBC patients treated with NAC. While systemic therapy and surgical resection had better OS for MpBC compared with patients who did not receive both systemic treatment and surgery and those unable to receive any curative-intent treatment, an adjuvant systemic approach was associated with superior survival for both the TN-MpBC and nTN-MpBC cohorts. On sensitivity analysis, this was further confirmed with OS for NAC patients only superseding systemic treatment approaches other than NAC in the pCR subset. Finally, on regression modeling in a propensity-score matched cohort of TN-MpBC to TN-IDC, NAC was independently associated with mortality compared with not undergoing NAC, as were clinical T4 and regional node-positive disease. These results challenge the perception that a TN-IDC-similar approach to treatment of MpBC is most appropriate and calls for metaplastic-specific treatment guidelines. Furthermore, use of NAC in MpBC should be considered with caution until factors associated with achieving pCR can be identified to determine patient-specific approaches to systemic therapy sequencing.

Of note, some advancements in systemic treatments for MpBC have been made. The low rate of pCR found in this study is an improvement from historic rates of < 10%.^33^ This may suggest improved effectiveness with an anthracycline- and taxane-based NAC regimen, which could result in increased rates of pCR and associated long-term benefits.^41^ Additionally, use of immunotherapy agents (namely anti‐CTLA4 blockade and anti‐PD‐1 blockade) has shown some promise, albeit at the risk of higher toxicity and mortality.^42^ Targeted therapies are also under current investigation for applicability in this cohort of breast cancers. One example is the combination of PI3K and MEK inhibitors, which have been demonstrated as effective in chemosensitive and chemoresistant xenograft models derived from MpBC patients.^43^ Finally, as an adjunct to systemic therapies and surgery, the importance of radiation therapy in MpBC appears to be distinct from that of other invasive breast cancers, as radiation therapy has been independently associated with OS and breast cancer-specific survival.^44^ As the outcomes of MpBC remain poor and well below that of TN-IDC with current application of surgery with or without sequencing of systemic therapies and radiation therapy, an approach to treatment distinct from that of TN-IDC is warranted. Clinical trials designed to investigate optimal treatment strategies for MpBC are needed along with updated guidelines for clinicians treating patients with MpBC.

This study has several limitations. Because this was a retrospective review of a large, national database, there is the potential for misclassification or miscoding. Any patients without complete data were excluded, which could have led to unknown bias in our results; however, the benefits of a large, multi-institutional database and the associated patient volume were felt to outweigh such risks. This is particularly true when investigating MpBC patients, as the disease’s rarity results in a lack of high-powered studies. Additionally, an inability to reference the source data limited the investigation to the provided data and made certain analyses impossible, such as the clinical decision making behind the choice of systemic therapy sequencing. Furthermore, outcome analysis afforded by the NCDB is limited to OS for patients for whom a recent follow-up has been documented. As such, cancer-specific outcomes based on systemic therapy strategies cannot be assessed. Any interpretation or application of OS in these cohorts should be interpreted with caution. Finally, the NCDB represents patients from only CoC‐accredited hospitals, and although these hospitals manage 70% of cancer cases, it may not accurately represent all national trends and practice patterns at non-CoC-accredited facilities.

## Conclusions

The results from this multi-institutional nationwide database study demonstrate that MpBC is predominantly treated with surgery and systemic therapy, with an increasing rate of NAC for triple-negative disease; however, patients with MpBC regardless of biomarker status have inferior survival to TN-IDC. Furthermore, a neoadjuvant approach for MpBC is associated with worse OS when compared with other systemic therapy sequences, unless a complete pathologic response is achieved. In correlation with previous studies, these findings reinforce the need for systemic treatment sequence optimization for MpBC.

## Supplementary Information

Below is the link to the electronic supplementary material.

Supplementary file1 (DOCX 37 KB)
