# Supplementary material for: The Metaplastic Conundrum: A National Cancer Database Analysis of Metaplastic versus Triple-Negative Ductal Breast Cancer
Source: Ann Surg Oncol. 2025 Jul 29;32(11):8448–60. doi: 10.1245/s10434-025-17932-3 (PMC12494619; doi:10.1245/s10434-025-17932-3)
Supplement: Supplementary file 1 — Supplementary file1 (DOCX 37 KB) [file 10434_2025_17932_MOESM1_ESM.docx]

**SUPPLEMENTARY MATERIALS**

**Supplemental Table 1: Baseline Characteristic Comparison Between Triple-Negative and Biomarker-Positive Metaplastic Breast Cancer.**

| Category  n (%) | Overall  n=5,575 |  | TN-MpBC  n=4,175 (74.9%) | nTN-MpBC  n=1,400 (25.1%) | p-value |
| --- | --- | --- | --- | --- | --- |
| Age, years (median [IQR]) | 62 [52-73] |  | 63 [53-73] | 61 [50-72] | **<0.0001** |
| Race |  |  |  |  | 0.99 |
| White | 4,285 (77.2%) |  | 3,211 (77.3%) | 1,074 (77.2%) |  |
| Black | 999 (18.0%) |  | 750 (18.1%) | 249 (17.9%) |  |
| Asian | 146 (2.6%) |  | 107 (2.6%) | 39 (2.8%) |  |
| Other | 115 (2.1%) |  | 86 (2.1%) | 29 (2.1%) |  |
| Ethnicity, Hispanic | 355 (6.5%) |  | 270 (6.6%) | 85 (6.2%) | 0.6 |
| Facility Type |  |  |  |  | **0.01** |
| Community Cancer Program (CCP) | 315 (6.3%) |  | 228 (5.8%) | 87 (6.7%) |  |
| Comprehensive CCP | 1,991 (38.1%) |  | 1,453 (37.0%) | 538 (41.4%) |  |
| Academic/Research Program | 1,746 (33.4%) |  | 1,344 (34.2%) | 402 (30.9%) |  |
| Integrated Network Cancer Program | 1,175 (22.5%) |  | 902 (23.0%) | 273 (21.0%) |  |
| Insurance Status |  |  |  |  | 0.09 |
| Not Insured | 108 (2.0%) |  | 79 (1.9%) | 29 (2.1%) |  |
| Private Insurance | 2,543 (46.0%) |  | 1,869 (45.2%) | 674 (48.4%)) |  |
| Government | 2,878 (52.0%) |  | 2,189 (52.9%) | 689 (49.5%) |  |
| Geographic Location Size |  |  |  |  | 0.5 |
| Metro | 4,770 (87.5%) |  | 3,566 (87.4%) | 1,204 (87.9%) |  |
| Urban | 603 (11.1%) |  | 460 (11.3%) | 143 (10.4%) |  |
| Rural | 79 (1.5%) |  | 56 (1.4%) | 23 (1.7%) |  |
| Distance from Treatment Facility, miles  (median [IQR]) | 9.3 [4.4-19.6] |  | 9.4 [4.5-19.8] | 9.1 [4.3-18.9] | 0.4 |
| Charlson-Deyo Comorbidity Index |  |  |  |  | 0.9 |
| 0 | 4,394 (78.8%) |  | 3,294 (78.9%) | 1,100 (78.6%) |  |
| 1 | 812 (14.6%) |  | 601 (14.4%) | 211 (15.1%) |  |
| 2 | 236 (4.2%) |  | 181 (4.3%) | 55 (3.9%) |  |
| ≥3 | 133 (2.4%) |  | 99 (2.4%) | 34 (2.4%) |  |

(nTN-MpBC: biomarker-positive metaplastic breast cancer, TN-MpBC: triple-negative metaplastic breast cancer)

**Supplemental Table 2: Baseline Characteristic Comparison Between Triple-Negative Metaplastic Breast Cancer, Biomarker-Positive Metaplastic Breast Cancer, and Triple-Negative Ductal Breast Cancer.**

| Category  n (%) | TN-MpBC  n=4,175 (2.8%) | nTN-MpBC  n=1,400 (0.9%) | TN-IDC  n=144,080 (96.3%) | p-value |
| --- | --- | --- | --- | --- |
| Age, years (median [IQR]) | 63 [53-73] | 61 [50-72] | 58 [49-68] | **<0.001** |
| Race |  |  |  | **<0.001** |
| White | 3,211 (77.3%) | 1,074 (77.2%) | 105,034 (72.9%) |  |
| Black | 750 (18.1%) | 249 (17.9%) | 31,842 (22.1%) |  |
| Asian | 107 (2.6%) | 39 (2.8%) | 4,034 (2.8%) |  |
| Other | 86 (2.1%) | 29 (2.1%) | 3,026 (2.1%) |  |
| Ethnicity, Hispanic | 270 (6.6%) | 85 (6.2%) | 9,941 (7.1%) | 0.3 |
| Facility Type |  |  |  | **<0.001** |
| Community Cancer Program (CCP) | 228 (5.8%) | 87 (6.7%) | 10,086 (7.0%) |  |
| Comprehensive CCP | 1,453 (37.0%) | 538 (41.4%) | 58,064 (40.3%) |  |
| Academic/Research Program | 1,344 (34.2%) | 402 (30.9%) | 30,833 (21.4%) |  |
| Integrated Network Cancer Program | 902 (23.0%) | 273 (21.0%) | 45,097 (31.3%) |  |
| Insurance Status |  |  |  | **<0.001** |
| Not Insured | 79 (1.9%) | 29 (2.1%) | 3,170 (2.2%) |  |
| Private Insurance | 1,869 (45.2%) | 674 (48.4%)) | 78,235 (54.3%) |  |
| Government | 2,189 (52.9%) | 689 (49.5%) | 62,675 (43.5%) |  |
| Geographic Location Size |  |  |  | 0.3 |
| Metro | 3,566 (87.4%) | 1,204 (87.9%) | 121,918 (86.6%) |  |
| Urban | 460 (11.3%) | 143 (10.4%) | 16,805 (11.9%) |  |
| Rural | 56 (1.4%) | 23 (1.7%) | 1,997 (1.4%) |  |
| Distance from Treatment Facility, miles  (median [IQR]) | 9.4 [4.5-19.8] | 9.1 [4.3-18.9] | 9.6 [4.7-20.0] | 0.07 |
| Charlson-Deyo Comorbidity Index |  |  |  | **<0.001** |
| 0 | 3,294 (78.9%) | 1,100 (78.6%) | 118,002 (81.9%) |  |
| 1 | 601 (14.4%) | 211 (15.1%) | 19,019 (13.2%) |  |
| 2 | 181 (4.3%) | 55 (3.9%) | 4,466 (3.1%) |  |
| ≥3 | 99 (2.4%) | 34 (2.4%) | 2,593 (1.8%) |  |

(nTN-MpBC: biomarker-positive metaplastic breast cancer, TN-MpBC: triple-negative metaplastic breast cancer, TN-IDC: triple-negative ductal breast cancer)

**Supplemental Table 3: Cox Proportional Hazard Regression of the One-to-One Propensity Score Matched Analysis.**

| Variable | Odds Ratio | 95% Confidence Interval | p-value |
| --- | --- | --- | --- |
| Age | 0.98 | 0.95, 1.01 | 0.2 |
| Race |  |  |  |
| White | Reference |  |  |
| Black | 0.57 | 0.26, 1.25 | 0.2 |
| Asian | 0.41 | 0.04, 3.82 | 0.4 |
| Other | 0.64 | 0.12, 3.55 | 0.6 |
| Ethnicity |  |  |  |
| Non-Hispanic | Reference |  |  |
| Hispanic | 0.71 | 0.20, 2.61 | 0.6 |
| Facility Type |  |  |  |
| Community Cancer Program (CCP) | Reference |  |  |
| Comprehensive CCP | 1.92 | 0.54, 6.90 | 0.3 |
| Academic/Research Program | 1.05 | 0.28, 3.98 | 0.9 |
| Integrated Program | 2.56 | 0.67, 9.85 | 0.2 |
| Insurance Status |  |  |  |
| Not Insured | Reference |  |  |
| Private Insurance | 0.64 | 0.14, 2.92 | 0.6 |
| Government | 1.23 | 0.26, 5.83 | 0.8 |
| Geographic Location Size |  |  |  |
| Metro | Reference |  |  |
| Urban | 1.62 | 0.61, 4.28 | 0.3 |
| Rural | 1.96 | 0.12, 33.09 | 0.6 |
| Distance from Treatment Facility, miles | 1.00 | 0.99, 1.01 | 0.7 |
| Histology |  |  |  |
| Triple-Negative Ductal | Reference |  |  |
| Triple-Negative Metaplastic | 0.54 | 0.11, 2.65 | 0.4 |
| Clinical Grade |  |  |  |
| Well Differentiated | Reference |  |  |
| Moderately Differentiated | 1.56 | 0.14, 17.04 | 0.7 |
| Poorly Differentiated/Undifferentiated | 1.17 | 0.12, 11.59 | 0.9 |
| Ki-67, % | 1.00 | 0.99, 1.01 | 0.9 |
| Lymphovascular Invasion |  |  |  |
| Not Present | Reference |  |  |
| Present | 0.74 | 0.34, 1.58 | 0.4 |
| Tumor Size, mm | 1.01 | 0.99, 1.03 | 0.4 |
| Clinical T Stage |  |  |  |
| cT1 | Reference |  |  |
| cT2 | 1.75 | 0.75, 4.06 | 0.2 |
| cT3 | 1.37 | 0.40, 4.76 | 0.6 |
| cT4 | 8.29 | 1.20, 57.00 | **0.03** |
| Clinical N Stage |  |  |  |
| cN0 | Reference |  |  |
| cN1 | 0.44 | 0.17, 1.13 | 0.09 |
| cN2 | 0.65 | 0.17, 2.52 | 0.5 |
| cN3 | 0.41 | 0.06, 2.74 | 0.4 |
| Extent of Surgical Resection |  |  |  |
| Partial Mastectomy | Reference |  |  |
| Mastectomy | 1.09 | 0.47, 2.52 | 0.7 |
| Bilateral Mastectomy | 0.81 | 0.29, 2.24 | 0.7 |
| Residual Tumor |  |  |  |
| No | Reference |  |  |
| Yes | 1.80 | 0.37, 8.85 | 0.5 |
| Regional Lymph Node Positivity |  |  |  |
| No | Reference |  |  |
| Yes | 6.53 | 1.24, 34.42 | **0.02** |
| Neoadjuvant Systemic Therapy |  |  |  |
| No | Reference |  |  |
| Yes | 2.56 | 1.36, 4.69 | **0.0003** |
| Received Chemotherapy |  |  |  |
| No | Reference |  |  |
| Yes | 2.11 | 0.00, ∞ | >0.99 |
| Received Immunotherapy |  |  |  |
| No | Reference |  |  |
| Yes | 0.72 | 0.14, 3.60 | 0.7 |
| Received Hormone Therapy |  |  |  |
| No | Reference |  |  |
| Yes | 0.00 | 0.00, ∞ | >0.99 |
| Received Radiation |  |  |  |
| No | Reference |  |  |
| Yes | 1.25 | 0.57, 2.75 | 0.6 |
| Pathologic T Stage |  |  |  |
| pT0 | Reference |  |  |
| pT1 | 8312900.82 | 0.00, ∞ | 0.9 |
| pT2 | 13450578.68 | 0.00, ∞ | 0.9 |
| pT3 | 14735614.28 | 0.00, ∞ | 0.9 |
| pT4 | 4267915.55 | 0.00, ∞ | 0.9 |
| Pathologic N Stage |  |  |  |
| pN0 | Reference |  |  |
| pN1 | 0.52 | 0.10, 2.74 | 0.4 |
| pN2 | 0.83 | 0.13, 5.26 | 0.8 |
| pN3 | 1.92 | 0.21, 17.46 | 0.5 |
